# Cariprazine and Lithium Overdose: A Case Report

**DOI:** 10.7759/cureus.112941

**Published:** 2026-07-19

**Authors:** Matthew C Tyler, Erin Woodward

**Affiliations:** 1 Intensive Care Unit, Gold Coast University Hospital, Southport, AUS; 2 Emergency Medicine, Eastern Health, Melbourne, AUS

**Keywords:** adult, cariprazine, lithium, overdose, toxicology

## Abstract

Cariprazine is a third-generation antipsychotic used in the treatment of schizophrenia and bipolar affective disorder. This case describes a significant overdose of cariprazine and lithium, detailing the clinical features and management outcomes.

A 24-year-old woman with a history of bipolar affective disorder and borderline personality disorder presented following an intentional overdose of approximately 60 mg of cariprazine and 3.75-5 g of lithium carbonate and ethanol. Her Glasgow Coma Scale score dropped from 15 to 3 briefly within three hours of ingestion, though she maintained airway patency and adequate oxygenation throughout. Serial electrocardiograms revealed no corrected QT prolongation. Serum lithium level peaked at 2.5 mmol/L six hours after ingestion. The patient was managed conservatively with intravenous fluids and close neurological monitoring and was fully alert and oriented within 12 hours. She was discharged without complications 48 hours after ingestion.

This appears to be the largest reported cariprazine overdose in an adult to date, although confounded by coingestion of ethanol and lithium. The primary clinical effect observed was transient sedation without respiratory compromise or cardiac dysrhythmia. Further evidence is needed, but this case suggests that even large overdoses of cariprazine may be managed conservatively in the absence of other complications, supporting a cautious but noninvasive approach in similar presentations.

## Introduction

Cariprazine is a third-generation antipsychotic used in the treatment of both acute and chronic psychoses as well as bipolar affective disorder, and was approved for use in Australia in 2020 [1-3]. Pharmacodynamically, cariprazine is distinguished from earlier generation antipsychotics by its partial agonist activity at the dopamine D2 and D3 receptors, with a notably higher (approximately 6-10-fold) binding affinity for D3 than D2, together with partial agonism at the serotonin 5-HT1A receptor and antagonism at 5-HT2A, 5-HT2B, and histamine H1 receptors [1,4,5]. This profile of higher affinity for D3 over D2 receptors has been proposed to underlie its favorable effect on negative and cognitive symptoms relative to older second-generation agents, while also contributing to a comparatively low burden of extrapyramidal side effects and metabolic disturbance [1,4]. It is a once-daily medication with an initial dose of 1.5 mg on commencement, and the typical maintenance daily dose is between 3 and 6 mg [6]. Despite widespread clinical use since regulatory approval, reports characterizing cariprazine toxicity in overdose remain scarce in adult populations, with only isolated cases described in the literature to date, most involving relatively modest or accidental ingestions [1]. This case details one of the largest patient-reported intentional overdoses of cariprazine, concurrently with lithium and ethanol, in the literature.

## Case presentation

A 24-year-old woman with a past medical history of post-traumatic stress disorder, personality affective disorder, bipolar affective disorder, anxiety, depression, overactive bladder syndrome, and previous episodes of self-harm and intentional overdose presented with a combined intentional overdose of 60 mg (10 tablets of 6 mg) of cariprazine and 3.75-5 g (15-20 tablets of 250 mg) of lithium carbonate in conjunction with one bottle of wine at 0530. The initial Glasgow Coma Scale (GCS) with the ambulance crew was 15, which then dropped to 10 (E2V3M5) at 0700 during triage [7]. Her GCS dropped to 3 at the time of medical officer review at approximately 0820, improving to 8 (E1V2M5) on repeat assessment after moving to a resus bay. She continued to maintain her airway patency and was monitored closely with peripheral oxygen saturation and end-tidal CO_2_ monitoring.

**Figure 1:**
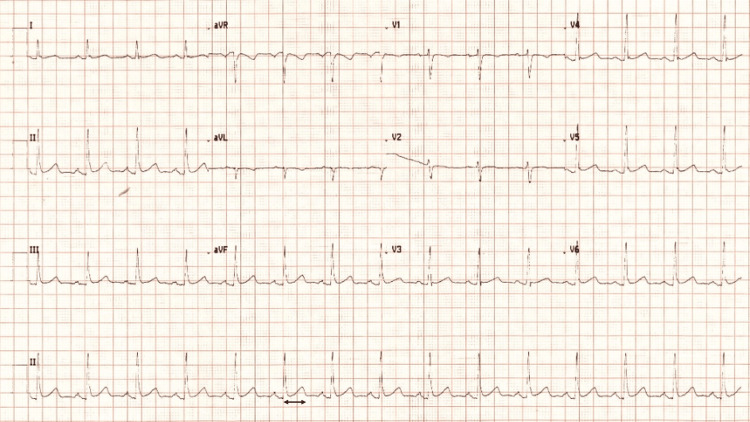
Admission ECG, demonstrating sinus rhythm with a ventricular rate of 87 bpm, PR interval of 160 ms, QRS of 92 ms, and QTc of 411 ms ECG: electrocardiogram; QTc: corrected QT; aVR: augmented vector right; aVL: augmented vector left; aVF: augmented vector foot

Initial observations showed a respiratory rate of 24 and an oxygen saturation of 98% on room air. Pulse rate was 88 bpm, and blood pressure was 118/80mmHg. Auscultation of the chest revealed clear and equal air entry. Her pupils were 4 mm and reactive to light. She had a normal tone in the upper and lower limbs. Her temperature was normal at 36.7°C. An oxybutynin patch found on her person was removed. Blood results from admission are detailed in Table 1. An electrocardiogram (ECG) on admission demonstrated normal sinus rhythm with a normal corrected QT (QTc) of 411 ms (Figure 1), with the longest QTc measured at 444 ms during admission.

**Table 1:** Admission and Day 1 blood results with reference ranges Hb: hemoglobin; WCC: white cell count; ALT: alanine aminotransferase; AST: aspartate aminotransferase; GGT: gamma-glutamyl transferase; ALP: alkaline phosphatase; pCO_2_: partial pressure of carbon dioxide

Parameter	Day 0	Day 1	Normal range
Hb (g/L)	159	138	115-155
Hematocrit (%)	45	40	33-45
WCC (10^9^/L)	6.5	6.9	4.0-12.0
Platelets (10^9^/L)	262	187	150-400
Sodium (mmol/L)	141	140	135-145
Potassium (mmol/L)	3.7	4.0	3.5-5.2
Urea (mmol/L)	2.9	2.4	2.8-8.1
Creatinine (mmol/L)	62	64	45-90
Bilirubin (mmol/L)	3	5	2-20
ALT (mmol/L)	60	40	5-35
AST (mmol/L)	30	21	5-30
GGT (mmol/L)	159	127	6-42
ALP (mmol/L)	78	59	30-110
Albumin (mmol/L)	41	30	34-47
Creatine kinase (mmol/L)	72	-	30-180
Venous pH	7.37	7.38	7.35-7.45
pCO_2_ (mmHg)	42	41	35-45
Glucose (mmol/L)	5.9	5.5	3.6-5.3
Lactate (mmol/L)	2.4	1.3	<2.2
Lithium level (mmol/L)	1	1.1	0.6-0.8
Paracetamol level (mmol/L)	<33	-	<33
Ethanol level (mmol/L)	26.3	-	0

The patient was commenced on intravenous 0.9% sodium chloride rehydration. A repeat lithium level taken six hours later was 2.5 mmol/L, and she was admitted for further rehydration and lithium level monitoring. She continued to have a fluctuating GCS over the next five hours, ranging from 3 to 15. At a later review, 10 hours after ingestion, she remained somnolent but easily arousable. At this time, she was able to mobilize to the bathroom with assistance. At 1800, approximately 11.5 hours, her GCS had returned consistently to 15.

She was discharged two days after admission; her lithium level was 0.5mmol/L, and other repeat blood results and ECG were unremarkable. She had no period of hypotension or pyrexia, and there was no evidence of akathisia or extrapyramidal symptoms throughout her admission.

## Discussion

There is limited published reporting of the effect of acute cariprazine overdose in adults. During the clinical trials, one significant accidental ingestion of 48 mg/day was reported, which resulted in orthostasis and sedation, with signs and symptoms resolving within the day [1]. There is some reporting of significant exposures within the pediatric population in the US [8]. They reported only 15% of cariprazine exposures had a moderate to major effect, with predominant symptoms being central nervous system depression and lethargy, and 75% of patients were discharged within three days [8]. They found the majority of moderate to major effects were observed at doses of 0.25-1.5 mg/kg [8]. Other case reports in adult populations typically describe adverse effects related to long-term use [9].

Cariprazine is metabolized into two main active components by hydroxylation and demethylation: desmethyl cariprazine and didesmethyl cariprazine [10]. Initial trial data in healthy volunteers demonstrated that the maximum plasma concentration of cariprazine and its active metabolites was 3.6 hours post-ingestion and up to 18.1 hours [10]. In the case described, the patient had a maximal depressed level of consciousness at approximately three hours after ingestion, although confounded by the coingestion of alcohol and lithium.

Akathisia and extrapyramidal symptoms are the main adverse events related to cariprazine, although often less frequently observed compared with first-generation antipsychotics [11,12]. Sedation is frequently seen in overdose with antipsychotics. Tachycardia, hypotension, and QTc prolongation may be cardiovascular features in antipsychotic overdose, while neuroleptic malignant syndrome (NMS) and seizures may be seen in severe cases [13]. In the case of this patient, only sedation was observed with no evidence of cardiovascular compromise, features of NMS, or movement disorders.

It was found that cariprazine had low dysrhythmogenic potential and did not cause QTc prolongation at three times the maximum recommended dose [10]. Indeed, this was also observed in this case with a normal QTc measured at every ECG during admission.

## Conclusions

This case report describes a significant combined overdose of 10 times the maximum daily dose of cariprazine in conjunction with lithium and alcohol. The findings of this case are similar to the presentation found during the clinical trials of significantly depressed level of consciousness, but essentially no other adverse effects. This case demonstrated that the main effect of this combined overdose was sedation without hypoventilation or loss of airway patency, and it had resolved by 12 hours after ingestion with no significant psychomotor effects or ECG changes. Reporting the direct effects of cariprazine in this case was limited, as this was a combined overdose and no confirmatory cariprazine serum level was taken. In this case, conservative supportive care without early endotracheal intubation was effective in the absence of airway compromise, but further evidence is needed to corroborate this approach to the management of cariprazine overdose.

## References

[1] Laszlovszky I, Barabássy Á, Németh G (2021). Cariprazine, a broad-spectrum antipsychotic for the treatment of schizophrenia: pharmacology, efficacy, and safety. Adv Ther.

[2] (2025). Therapeutic goods administration (TGA): reagila. https://www.tga.gov.au/resources/auspmd/reagila.

[3] (2025). Australian medicines handbook: cariprazine. https://amhonline.amh.net.au.acs.hcn.com.au/chapters/psychotropic-drugs/antipsychotics/cariprazine.

[4] Citrome L (2013). Cariprazine: chemistry, pharmacodynamics, pharmacokinetics, and metabolism, clinical efficacy, safety, and tolerability. Expert Opin Drug Metab Toxicol.

[5] Kiss B, Horváth A, Némethy Z (2010). Cariprazine (RGH-188), a dopamine D(3) receptor-preferring, D(3)/D(2) dopamine receptor antagonist-partial agonist antipsychotic candidate: in vitro and neurochemical profile. J Pharmacol Exp Ther.

[6] Citrome L (2015). The ABC's of dopamine receptor partial agonists - aripiprazole, brexpiprazole and cariprazine: the 15-min challenge to sort these agents out. Int J Clin Pract.

[7] Teesdale J, Graham B (1974). Assessment of coma and impaired consciousness: a practical scale. Lancet.

[8] Husak N, Laudone TW, Leonard JB (2023). A descriptive study of aripiprazole, brexpiprazole, and cariprazine exposures in children ages 0 to 5 years reported to United States poison centers. Clin Toxicol (Phila).

[9] Heck J, Seifert J, Stichtenoth DO (2021). A case series of serious and unexpected adverse drug reactions under treatment with cariprazine. Clin Case Rep.

[10] McCormack PL (2015). Cariprazine: first global approval. Drugs.

[11] Barabássy Á, Sebe B, Acsai K, Laszlovszky I, Szatmári B, Earley WR, Németh G (2021). Safety and tolerability of cariprazine in patients with schizophrenia: a pooled analysis of eight phase II/III studies. Neuropsychiatr Dis Treat.

[12] Lao KS, He Y, Wong IC, Besag FM, Chan EW (2016). Tolerability and safety profile of cariprazine in treating psychotic disorders, bipolar disorder and major depressive disorder: a systematic review with meta-analysis of randomized controlled trials. CNS Drugs.

[13] Minns AB, Clark RF (2012). Toxicology and overdose of atypical antipsychotics. J Emerg Med.

